# Sedentary behavior, abdominal obesity and healthcare costs in Brazilian adults with cardiovascular diseases: a cross-sectional study

**DOI:** 10.1590/1516-3180.2023.0029.140823

**Published:** 2023-12-11

**Authors:** Maria Carolina Castanho Saes Norberto, Monique Yndawe Castanho Araujo, Suelen Jane Ricardo, Charles Rodrigues, Juziane Teixeira Guiça, Bruna Camilo Turi-Lynch, Jamile Sanches Codogno

**Affiliations:** I MD. Master’s Student, Physical Education, Universidade Estadual Paulista (UNESP), Presidente Prudente (SP), Brazil.; II PhD Professor, Post-graduate Program in Movement Sciences, Universidade Estadual Paulista (UNESP), Presidente Prudente (SP), Brazil.; III MSc, Physical Education, Post-Graduate Program in Physiotherapy, Universidade Estadual Paulista (UNESP), Presidente Prudente, Brazil.; IV MD. Master’s Student, Physical Education, Universidade Estadual Paulista (UNESP), Presidente Prudente (SP), Brazil.; V MD. Master’s Student, Physical Education, Universidade Estadual Paulista (UNESP), Presidente Prudente (SP), Brazil.; VI PhD. Professor, Department of Physical Education and Exercise Science, Lander University, Greenwood, South Carolina, United States of America.; VII PhD. Professor, Post-Graduation Program in Movement Sciences, Post-graduate program in Physiotherapy, Universidade Estadual Paulista (UNESP), Presidente Prudente (SP), Brazil.

**Keywords:** Sedentary behavior, Public health, Health care costs, Fat body, Waist circumference, Brazil, Chronic disease, Medicines, Adult patients

## Abstract

**BACKGROUND:**

Research on the economic burden of sedentary behavior and abdominal obesity on health expenses associated with cardiovascular diseases is scarce.

**OBJECTIVE:**

The objective of this study was to verify whether sedentary behavior, isolated and combined with abdominal obesity, influences the medication expenditure among adults with cardiovascular diseases.

**DESIGN AND SETTING:**

This cross-sectional study was conducted in the city of President Prudente, State of São Paulo, Brazil in 2018.

**METHODS:**

The study included adults with cardiovascular diseases, aged 30-65 years, who were treated by the Brazilian National Health Services. Sedentary behavior was assessed using a questionnaire. Abdominal obesity was defined by waist circumference. Medication expenditures were verified using the medical records of each patient.

**RESULTS:**

The study included a total of 307 adults. Individuals classified in the group with risk factor obesity combined (median [IQ] USD$ 29.39 [45.77]) or isolated (median [IQ] USD$ 27.17 [59.76]) to sedentary behavior had higher medication expenditures than those belonging to the non-obese with low sedentary behavior group (median [IQ] USD$ 13.51 [31.42]) (P = 0.01). The group with combined obesity and sedentary behavior was 2.4 (95%CI = 1.00; 5.79) times more likely to be hypertensive.

**CONCLUSION:**

Abdominal obesity was a determining factor for medication expenses, regardless of sedentary behavior, among adults with cardiovascular diseases.

## INTRODUCTION

The use of medicines has been the basis of many clinical interventions to treat a large variety of diseases and represents one of the most relevant components of overall healthcare costs. Healthcare costs related to medicine use increases with age^1,2^ and represents a relevant challenge for the management of any national health service.

The prevalence of sedentary behavior and abdominal obesity has increased worldwide, and these phenomenon seems to have been boosted by the coronavirus pandemic.^3^ Even before the pandemic, the relevant burden of both abdominal obesity and sedentary behavior on the development of cardiovascular and metabolic diseases has been reported by several authors.^4,5,6,7^ Part of this attention directed to abdominal obesity and sedentary behavior is because the diseases associated with these factors put relevant pressure on national health services worldwide.

One hour of sedentary behavior can add up to approximately USD $37 in personal health expenditures.^8^ It has been defined as activities that do not increase energy expenditure substantially above the resting level and involves energy expenditure of 1.0 to 1.5 metabolic equivalent units (METs).^9^ Sedentary behavior, over the last decade, has been associated with numerous chronic non-communicable diseases (NCDs).^10-12^

An epidemiological study conducted over a span of 12 years on individuals aged 18-90 years in Canada showed that spending excessive time on sedentary behaviors can have a negative impact on various health outcomes, regardless of the individual’s physical activity level. Those who reported spending approximately three-quarters of their time, or almost all of their time throughout the day, sitting (hazard ratio, HR = 1.47 [95% confidence interval (CI) = 1.09–1.96]; HR = 1.54 [95%CI = 1.09–2.17]) were at a higher risk for cardiovascular disease-associated mortality when compared to those who reported almost no time sitting.^13^

Obesity has been associated with cardiovascular disease-associated mortality (HR = 1.50, 95%CI = 1.08; 2.08).^14^ In addition to the economic burden that ranges between 0.7%-2.8% of a country’s total health budget,^15^ evidence shows that abdominal obesity increases the probability of higher medication expenditure by 1.66 times.^16^

Obesity has been associated with economic losses in the public and private sectors.^17^ However, although sedentary behavior is widely associated with a large variety of health outcomes, its economic impact remains unclear. Moreover, even when related to each other (obesity and sedentary behavior), the combined impact of both on healthcare costs has barely been investigated, mainly in developing nations, the home of most of the world’s population.

We hypothesized that the combination of obesity and sedentary behavior impacts the costs attributed to medication. The findings of this study would be useful in motivating stakeholders to prioritize investments in the prevention of these two risk factors (especially sedentary behavior), which would aid in mitigating the healthcare costs.

There is evidence in the literature on how sedentary behavior affects health,^14^ but information regarding its impact on economics and healthcare costs is scarce. Moreover, research that explores the economic burden of sedentary behavior and obesity, in aggregate form, on healthcare expenditures associated with cardiovascular disease is also scarce. It is believed that the presence of both risk factors maximizes health expenditures.

## OBJECTIVE

The objective of this study was to verify whether sedentary behavior, isolated and combined with abdominal obesity, influences medication expenditure among adults with cardiovascular diseases.

## METHODS

### Study population

This study presents a descriptive research model and involves cross-sectional evaluation of participants along with a longitudinal cost analysis. These results refer to the first data collection (baseline) of an ongoing cohort study conducted in the city of Presidente Prudente (with approximately 230,000 inhabitants), located in the western region of the State of São Paulo, Brazil.

Patient selection was carried out through the medical records of the Regional Hospital, which offers referral care of medium and high complexity, totally free of charge, to 45 cities and municipalities in the western region of the state, with an average turnover of 447.36 patients/day.

The minimum sample size was calculated taking into consideration the annual number of patients treated at the Regional Hospital (n = 163,288) as well as the number of patients (aged 30-65 years) treated for cardiovascular reasons (Category I of the International Classification of Diseases and Related Health Problems [ICD] [~ 0.74%, n = 1,200]). Thus, considering a percentage of 0.74%, sampling error of 5%, and Z = 1.96, the minimum sample size was estimated to be 106. Finally, by adding an estimated loss of 100% throughout the follow-up period (estimated from previous studies), a minimum of 212 participants were required to participate in this study.

Participants were randomly selected using medical records from the cardiology department (last six months) of the Regional Hospital. After the selection of patients from the records, it was verified whether they met the following inclusion criteria: i) age ranging between 30-65 years (age group with a high prevalence of chronic diseases in Brazil);^18^ ii) use of the services offered by the Brazilian National Healthcare System for cardiovascular diseases in the last year; and iii) residing in the city of Presidente Prudente. Researchers could obtain information regarding the use of primary healthcare services. Patients were excluded if they: i) did not meet at least one inclusion criterion; ii) were deceased; iii) had an inactive phone number; and iv) missed at least two scheduled appointments for data collection.

The selected patients were contacted via telephone and invited to participate in face-to-face interviews and evaluations (conducted in July and August 2018). Patients who agreed to participate in the study signed a consent form.

**Figure 1:**
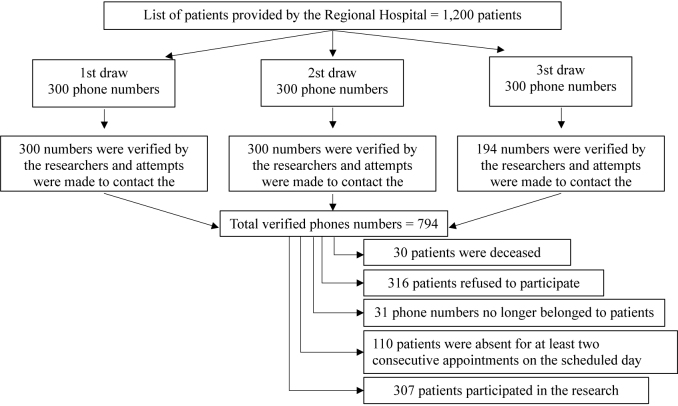
Flow chart depicting the selection of the study population.

From the list of 1,200 patients provided by the Regional Hospital, random draws in blocks (300 patients per draw) were performed using STATA software version 16.0 (StataCorp LLC, College Station, Texas, USA) (**Figure 1**).

All telephone numbers selected in the first and second draws (n = 600) were verified by the researchers and five attempts were made to contact the patients. A third draw was required, and 194 patients were contacted until the minimum sample size was reached (**Figure 1**). Among the 794 patients contacted, 307 agreed to participate in the study, 316 declined to participate, 31 telephone numbers no longer belonged to the patient, 30 belonged to deceased patients, and 110 missed at least two scheduled appointments for data collection (**Figure 1**).

### Ethical Considerations

The study design and methodology was approved on May 22, 2018, by the Ethics Research Committee of São Paulo State University (Protocol number CAAE 82767417.5.0000.5402). The study was conducted in accordance with the tenets of Declaration of Helsinki and informed consent was obtained from all the participants prior to the commencement of the study.

### Dependent Variables

#### Medication expenditures

Estimated expenditures refer to medications used by patients in primary healthcare. Medication expenditures were estimated, including information registered in medical records 12 months prior to the date of face-to-face evaluation (July/August 2017 to July/August 2018).^2,19^

Medication expenditures were calculated by multiplying the number of medications with the price and daily quantity. Prices of medication distributed to the patient (funded by the Brazilian National Healthcare System) were based on information from standard tables for reimbursement of services provided to the municipal government for the year of purchase. Monetary values were expressed in Reais (R$) and updated in accordance with the official Brazilian inflation index (Extended National Consumer Price Index, IPCA), from the date of obtaining the data until December 2022, and converted into US dollars (US$) using the official exchange rate of the same date (dollar exchange rate at 5.21) published by the Brazilian Central Bank.^20^

#### Presence of NCDs

Information regarding chronic diseases such as arterial hypertension, hypercholesterolemia, diabetes mellitus, heart attack, atherosclerosis, and nephritis, was first obtained through medical records in the sampling process and then verified via interview using a questionnaire.^21^ The interviewee reported the following: (i) diagnosis of the disease; and (ii) use of medications.

### Independent variables

#### Sedentary behavior and abdominal obesity

Sedentary behavior was assessed using a questionnaire developed by Mielke et al.^22^ The instrument included questions regarding time spent on sedentary behavior (activities such as watching television, using a computer, and remaining seated) on a typical weekday in different environments: i) work; ii) educational setting (school or college/university); iii) transportation (car, bus, and motorcycle); and iv) home. This instrument was submitted to test-retest reliability study and the intraclass correlation coefficients and Lin concordance score were ≥ 0.7 for all items and total score.^19^

For the present study, participants were classified according to daily time (hours) spent on sedentary behavior: i) high sedentary behavior (HSB) ≥ 8 h, and ii) low sedentary behavior (LSB) < 8 h. This cutoff point was adopted based on a study that included a similar population and found that HSB (≥ 8 h per day) was associated with higher all-cause mortality risk.^23^

Abdominal obesity was defined by waist circumference (WC), with cutoff points being 102 cm for men and 88 cm for women.^24^

For statistical analysis, a new variable was created considering the cluster of sedentary behavior and abdominal obesity, resulting in three groups: i) HSB and abdominal obesity (Obese + HSB); ii) HSB or abdominal obesity (Intermediate [Obese + LSB or Non-obese + HSB]); and iii) LSB and no abdominal obesity (Non-obese + LSB).

#### Adjustment variables and patient characterization

Sex and age of the participants were recorded during the interview. Economic condition (EC) was verified according to the patient’s monthly income.^25^ These were considered confounding variables due to their association with chronic disease diagnosis.

Weight and the percentage of body fat were measured using bioelectrical impedance (InBody brand model 230, InBody Co., Seoul, South Korea). Height was measured during the interview using Sanny Caprice stadiometer (ES2060, Sanny, Sao Paulo, Brazil). Diastolic and systolic blood pressures were measured using a manual device (BIC brand APO336, CBMED, Itupeva, Brazil.) according to the Brazilian Guideline of Arterial Hypertension.^26^

### Statistical analysis

Normality of data was verified using the Kolmogorov-Smirnov test, and further analyses were performed according to the distribution of the dataset. Descriptive statistics were presented as mean values, standard deviation (SD), median, interquartile range (IQ), and 95%CI for numerical variables, and as percentage values for categorical variables. Comparisons between groups were verified using the analysis of variance (ANOVA) test (with Tukey’s post hoc) and the Kruskal-Wallis test (with Mann-Whitney as post hoc) when the variables were normal and not normal, respectively. Associations between categorical variables (presence of chronic diseases and the cluster of sedentary behavior and abdominal obesity) were tested using the chi-square test, and when significant, the magnitude of the associations was expressed as OR and its 95%CI using binary logistic regression. Statistical significance (P value) was set at 5%, and all analyses were performed using STATA 16.0 statistical software (Stata LLC, Texas, United States).

## RESULTS

The study included a total of 307 adults with cardiovascular diseases. The mean age of the study population was 54.38 (8.29) years, and it comprised 160 (52.1%) men and 147 (47.9%) women. Regarding the level of education, 5.2% of the participants (n = 16) had a college degree, 27% (n = 83) had completed high school, 46.6% (n = 143) had completed elementary school, and 21.2% (n = 65) had not completed elementary education. All participants were classified as having low EC (< R$ 5,000.00 per month, USD$ 1,225.04).

The prevalence of HSB and abdominal obesity in the study population was 22.1% (n = 68) and 65.1% (n = 200), respectively. The general characteristics of the study participants are presented in **Table 1**. Differences were observed among the groups in terms of age, height, weight, body mass index (BMI), WC, and systolic blood pressure (P < 0.05).

**Table 1:** General characteristics of the study population in terms of the three groups studied

Variable	Non-obese + LSB (n = 93) Mean (SD)	Intermediate (n = 160) Mean (SD)	Obese + HSB (n = 54) Mean (SD)	P value*
Age (years)	52.51 (8.94)	55.54 (7.67)^a^	54.13 (8.46)	**0,019**
Height (cm)	158.16 (7.17)	164.33 (9.50)^a^	169.51 (8.0)^a,b^	**0,001**
Weight (kg)	66.39 (9.88)	86.55 (16.32)^a^	92.06 (17.25)^a^	**0,001**
BMI (kg/m²)	26.65 (4.33)	32.08 (5.52)^a^	31.92 (5.40)^a^	**0,001**
WC (cm)	87.86 (8.72)	106.12 (13.12)^a^	107.50 (14.15)^a^	**0,001**
%BF (%)	34.81 (9.63)	37.58 (9.67)	35.70 (8.20)	0,070
DBP (mm/Hg)	85.38 (76.10)	84.47 (13.48)	84.34 (15.99)	0,985
SBP (mm/Hg)	118.06 (19.90)	127.86 (19.20)^a^	127.17 (19.64)^a^	**0,001**
Sum of diseases	2.00 (1.89)	2.38 (1.66)	2.28 (1.74)	0,170

* P < 0.05 for the One-Way ANOVA; ^a^ significant difference (P < 0.05) when compared to the non-obese + LSB group (Tukey Post Hoc test); ^b^ significant difference (P < 0.05) when compared to the Intermediate group (Tukey Post Hoc test); LSB = Low Sedentary Behavior; HSB = High Sedentary Behavior; SD = standard deviation; cm = centimeters; kg = kilograms; BMI = body mass index; kg/m² = kilograms per square meter; WC = waist circumference; %BF = percentage of body fat; DBP = diastolic blood pressure; SBP = systolic blood pressure; mm/Hg = millimeters of mercury.

**Figure 2:**
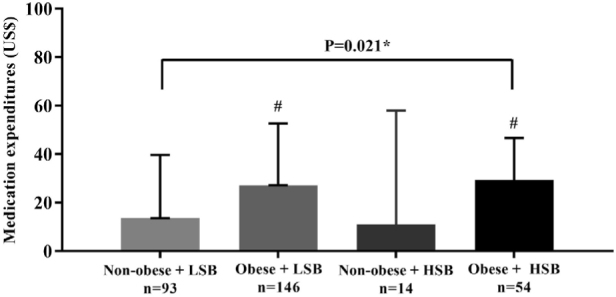
Medication expenditures according to the cluster of sedentary behavior and abdominal obesity.

When comparing medication expenditures according to sedentary behavior and abdominal obesity grouping, we found that individuals classified in the obese + HSB group had higher expenses than those in the non-obese + LSB group (median [IQ] USD$ 29.39 [45.77] versus USD$ 13.51 [31.42]; P = 0.01). Among those classified in the intermediate group, it was observed that those who were only obese (obese + LSB) had higher expenses than the Non-obese + LSB group (median [IQ] USD$ 27.17 [59.76] versus USD$ 13.51 [31.42]; P = 0.05). However, the same was not observed for those with only HSB (Non-obese + HSB) (median [IQ] USD$ 11.04 [63.54] versus USD$ 13.51 [31.42]; P = 0.97) (**Figure 2**).

LSB = Low Sedentary Behavior; HSB = High Sedentary Behavior; * P < 0.05 for Kruskal-Wallis test; # = significant difference (P < 0.05) when compared to the non-obese + LSB group (Mann-Whitney test).

When analyzing the association between chronic diseases and the cluster of sedentary behavior and abdominal obesity, we found significant results for arterial hypertension (P = 0.004) and heart attack (P = 0.035). Binary logistic regression analysis showed that individuals with abdominal obesity and HSB were 2.4 times more likely to be hypertensive than non-obese + LSB individuals. Age was a significant risk factor in this model for hypertension (OR = 1.07 [95%CI = 1.04; 11.11]) and heart attack (OR = 1.05 [95%CI = 1.01; 1.08]) (**Table 2**).

**Table 2:** Association between presence of chronic diseases and the cluster of sedentary behavior and abdominal obesity

NCDs	%(n)	P value	OR [95%CI]	H-l P value
**Arterial Hypertension**		**0.004**		0.843
Non-obese + LSB	53.8(50)		1.00	
Intermediate	73.1(117)		1.71 (0.92; 3.19)	
Obese + HSB	74.1(40)		**2.40 (1.00; 5.79)**	
**Hypercholesterolemia**		0.140		
Non-obese + LSB	34.4(32)		---	
Intermediate	41.3(66)		---	
Obese + HSB	46.3(25)		---	
**DM**		0.100		
Non-obese + LSB	19.4(18)		---	
Intermediate	24.4(39)		---	
Obese + HSB	31.5(17)		---	
**Heart attack**		**0.035**		
Non-obese + LSB	22.6(21)		1.00	0.731
Intermediate	37.5(60)		1.01 (0.51; 1.97)	
Obese + HSB	37.0(20)		0.88 (0.37; 2.09)	
**Atherosclerosis**		0.394		
Non-obese + LSB	17.2(16)		---	
Intermediate	22.5(36)		---	
Obese + HSB	22.2(12)		---	
**Nephritis**		0.500		
Non-obese + LSB	7.5(7)		---	
Intermediate	4.4(7)		---	
Obese + HSB	5.6(3)		---	

* = P < 0.05 for chi-square test followed by binary logistic regression; OR = Odds ratio (OR adjusted for sex, age, and educational level); HSB = high sedentary behavior; LSB = low sedentary behavior.

In addition, it was found that individuals with hypertension had higher expenditures for medication (median [IQ] USD$ 33.85 [55.03] versus USD$ 4.83 [21.69]) when compared with normotensive patients (P = 0.01).

## DISCUSSION

Our study population comprised Brazilian adults with cardiovascular diseases. Our primary finding was that the groups that had higher expenses for medication had abdominal obesity risk factor, and when abdominal obesity and HDB were combined, a robust association with prevalence of arterial hypertension was observed.

We found that 22.1% of the study participants had HSB. This proportion was not very different from the estimates of the World Health Organization study which included six low- and middle-income countries, and reported that sedentary behavior varied from 21%-58% among the adult population.^27^ Similarly, a Brazilian study showed that approximately 30% of the adults (≥ 20 years) reported 3 h/day of sedentary behavior, while approximately 20% reported sedentary behavior spanning 6h/day-9h/day. On an average, the participants reported spending 5.8 (SD 4.5) h/day sitting.^22^

Additionally, 65.1% of our study population presented with abdominal obesity. This was similar (approximately 70%) to the proportion reported by a previous research (approximately 70%) with a comparable population.^16^ In our study, 17.6% participants had a cluster of sedentary behavior and abdominal obesity. Literature has shown that the likelihood of obesity is 3.21 times higher among sedentary individuals.^28^

The present study showed higher medication expenses for individuals classified in the groups with obesity risk factor (Obese + LSB and Obese + HSB), indicating that this variable was a determinant of medication expenses. The relationship between obesity and healthcare expenditure has been well explored by several previous studies.^16,29,30^ An Australian study showed that higher obesity rates correlated with higher expenditures, with costs being 19%-51% higher in comparison to individuals with normal weight.^29^

A Brazilian study reported that an increase in the number of obese individuals in a household was proportional to the increase in healthcare expenditures (P < 0.001), especially in the context of medications.^27^ Additionally, among the population assisted by the primary healthcare system, it was observed that medication expenditure represented 35.2% of all expenditures related to health services. Moreover, it has been reported that increased WC and low level of physical activity were related to higher medication expenditures (rho = 0.25, P value = 0.001 and rho = -0.13, P value = 0.001).^16^

**Figure 2** show higher expenses for medication when HSB was combined with obesity. However, the group with isolated HSB did not appear to have significantly higher expenses than the Non-obese + LSB group. The total healthcare costs attributable to sedentary behavior in 2016-2017 in the United Kingdom was £ 800 million. In addition, cardiovascular disease costs attributable to sedentary behavior reached £ 424 million (£ 367 to £ 480 million), followed by £ 281 million (£ 233 to £ 327 million) for diabetes.^31^ In Finland, healthcare costs attributable to sedentary behavior (≥ 8 h/day) totaled approximately € 1.5 billion in 2017.^32^

To the best of our knowledge, this is one of the first study to describe the potentially harmful impact of sedentary behavior combined with obesity on healthcare costs in developing nations. Therefore, contextualizing the values presented in this study, it is worth noting that individuals who were obese and had HSB spent 12.6% of the national minimum wage on medicines (quotation referring to December 2022 [USD$ 232.6]; 9.65% of average per capita income in Brazil in 2022 [USD$ 304.4]).^33^

The economic impact of the combination of sedentary behavior and obesity can be linked to the onset of NCDs. An Australian study including more than 8,000 adults showed a negative association between sedentary behavior and mortality due to cardiovascular diseases (risk ratio = 1.18, 95%CI = 1.03, 1.35).^34^ Obese individuals have a tendency to develop cardiovascular diseases,^35^ such as arterial hypertension,^36^ due to metabolic dysfunctions, which may promote insulin resistance^37^ and consequently result in coronary microvascular dysfunction.^38^

Studies suggest that hypertension is more likely to occur in people with excess weight and sedentary lifestyle (OR = 4.09, 95%CI = 1.93–8.63) or with abdominal obesity and sedentary lifestyle (OR = 4.69, 95%CI = 2.35–9.35) when compared to individuals with normal weight and active lifestyle.^39^ We found that sedentary behavior and abdominal obesity increased the likelihood of being diagnosed with arterial hypertension by 2.4 times, a fact that can justify the increase in medication expenditure. Studies in the United States^40-42^ have reported that individuals with hypertension spend 6.42 times more on medications in comparison to normotensive individuals (P < 0.001),^40^ and that annual medical expenses associated with hypertension has increased significantly by 8.3% (P = 0.015).^41^ In Canada, hypertension accounts for 10.2% of the total health expenditure, $ 13.9 billion in 2010 and projections estimate $ 20 billion by 2020.^42^ In Brazil, hypertension is one of the three cardiovascular diseases that imposes high expenditure on the universal health system.^43^

In our study, other relevant diseases, such as diabetes mellitus, atherosclerosis, and dyslipidemia, were not found to be significantly associated with sedentary behavior and abdominal obesity. A potential explanation for this may be an underestimation of the actual prevalence of these diseases in our study population. In fact, all of these disease entities require more complex diagnostic methods than arterial hypertension and heart attack.

A possible non-medical alternative to prevent and minimize health expenditures would be to strengthen public health programs with a focus on healthy lifestyle through physical activity and reduction of risk factors such as obesity. It has been reported that every minute of physical activity can reduce the odds of abdominal obesity by 4% and 2% in men and women, respectively.^28^

Evidence suggests that physical activity promotes numerous health benefits, such as decreased incidence of all-cause mortality, cardiovascular diseases, cancer, and diabetes. Performing physical activity of any sort is recommended for all age groups and is better than doing none. At the same time, it is recommended that sedentary behavior be replaced by physical activity, even that of light intensity.^44^

Therefore, we emphasize the importance of our findings, which would be useful for policymakers when allocating health resources to public health programs targeting risk factors such as obesity and sedentary behavior. Furthermore, future research is important to elucidate the complex relationships between sedentary behavior and health outcomes.

The main limitation of this study was reverse causality due to its cross-sectional design. In addition, a questionnaire was used instead of accelerometers to evaluate sedentary behavior. Moreover, the analyses carried out did not allow the assessment of the burden of each isolated disease, not even the one that had the greatest impact on health expenditure. Sensitivity analyses were not performed. It must also be considered that the prevalence of some diseases may have been underestimated in our sample, limiting the power of associations tested. Finally, the expenditure on medications included in the present study represents only a part of the expenses of these patients, since they could have used medications paid for from their own budget. Moreover, this could also have been the case in terms of use of other healthcare systems (e.g., tertiary and secondary care). However, we have highlighted the importance of our findings in the context of public health, revealing the burden of sedentary behavior and abdominal obesity on the public health system.

## CONCLUSION

Abdominal obesity proved to be a determining factor for medication expenses, regardless of sedentary behavior, among adults with cardiovascular diseases.
